# Levels of potentially toxic and essential elements in Tocantins River sediment: health risks at Brazil’s Savanna-Amazon interface

**DOI:** 10.1038/s41598-024-66570-4

**Published:** 2024-08-04

**Authors:** Thiago Machado da Silva Acioly, Marcelo Francisco da Silva, José Iannacone, Diego Carvalho Viana

**Affiliations:** 1 Postgraduate in Animal Science (PPGCA/UEMA), Multi-User Laboratories in Postgraduate Research (LAMP), State University of Maranhão, São Luís, 65081-400 Brazil; 2 Center for Exact, Natural and Technological Sciences (CCENT), State University of the Tocantina Region of Maranhão (UEMASUL), Imperatriz, 65900-000 Brazil; 3 Animal Ecology and Biodiversity Laboratory (LEBA), Universidad Nacional Federico Villarreal, 15007 Lima, Peru; 4 Center of Agrarian Sciences, Center for Advanced Morphophysiological Studies (NEMO), State University of the Tocantina Region of Maranhão (UEMASUL), Imperatriz, 65900-000 Brazil

**Keywords:** Aquatic toxicology, Biomonitoring, Ecotoxicology, Environmental impact, Heavy metals, Environmental impact, Freshwater ecology

## Abstract

The field study aims to address identified research gaps by providing valuable information on the concentration, spatial distribution, pollution levels, and source apportionment of toxic and essential elements in sediment samples from four sampling sites (P1: Beira Rio (urban area), P2: Bananal (rural area), P3: Embiral (rural area), P4: Cidelândia (rural area) distributed along the middle Tocantins River, Brazil. Samples were collected in 2023 from river sections and analyzed using various contamination índices (geoaccumulation index, contamination factor, enrichment factor, pollution load index, sediment pollution index, potential ecological risk coefficients, and integrated risk index). Results indicated that the levels of aluminum, iron, manganese, and selenium exceeded legal standards in that year. Chromium, nickel, copper, zinc, and lead exceeded guidelines, mainly in section P1 for aluminum and section P3 for nickel and lead. Rainy months showed increased presence, indicating seasonal variability. The geoaccumulation index indicated low pollution levels, with lead and nickel notably present near urban and industrial areas. The enrichment factor highlighted elevated concentrations of lead and zinc in industrial areas. Both PLI and SPI indices raise concerns regarding Pb (P4) and Zn (P3) concentrations at specific times of the year. Overall, potential ecological risks were deemed low for most sites. Continuous monitoring and interventions are crucial to preserve water and environmental quality in the region.

## Introduction

The aquatic environment faces constant threats from contamination due to various anthropogenic pollutants, which stem from different industrial, agricultural, and urban activities. These pollutants can have diverse origins and compositions, depending on the specific activities occurring in a given region^1^. For instance, mining, agrochemicals, and industrial wastewater contribute to sediment heavy metal contamination^2,3^. Urban wastewater discharge may contain high levels of Zn, Pb, and Cu^4^ (Ancieta 2012).

Sediments serve a crucial role in preserving the ecological status of water bodies, functioning as essential ecological components in aquatic reservoirs^5^. However, despite their significance, sediments also act as repositories for various pollutants, especially heavy metals, significantly contributing to the mobilization of contaminants within aquatic systems under favorable conditions^6,7^. Acknowledging their importance as hosts for trace metals, it is imperative to integrate sediments into environmental monitoring programs^8^.

Studies highlight the contamination of potentially toxic elements as a severe ecological crisis in aquatic environments due to their destructive, non-degradable, and bioaccumulative impact on organisms throughout the food web^9^, Padhye et al.^10,11^. While essential elements like Na, Ca, Mn, Zn, and Fe are vital for organisms in trace amounts (Amin et al. 2021), non-essential or toxic elements like Cd, Hg, and Pb pose significant risks, even at minimal concentrations^12,13^. Additionally, these elements strongly adhere to suspended sediments, leading to their rapid deposition in bed sediments and detrimental effects on aquatic diversity^14^. In cases of strong sorption, suspended particle settling and sediment formation scavenge contaminants from the water phase, contributing to their accumulation in river and lake beds^15^.

The quality of sediments serves as a crucial indicator of water pollution, providing valuable insights into pollutant levels. Sediment contamination, as emphasized by Alsamadany et al.^16^, is a significant factor in degrading aquatic environments, leading to the development of various monitoring and management methods. Monitoring sediment quality is essential for assessing water pollution, and it's imperative to share this information with the community and government to develop plans for safeguarding valuable freshwater resources. Only through such measures can we effectively control and prevent damage to the balance of the aquatic ecosystem and the health of the population.

Various indices can be used to assess potential metal pollution levels in sediment. It's important to note that all risk assessment methods provide theoretical, probabilistic results^17^. These indices are widely employed for detecting metal contamination and are firmly established in the literature, often mandated by legislation^18^. For instance, the Pollution Load Index (PLI) offers a comprehensive assessment of sediment quality regarding metal pollution, aiding in categorizing pollution levels based on established criteria. Spatial analyses of metal levels in sediments, along with differentiation from natural backgrounds, are essential and can help recognize the methods of accumulation and geochemical behavior of metals in aquatic ecosystems.

The Tocantins River, also known as the Araguaia-Tocantins due to its mouth in Pará, flows through the states of Maranhão and Tocantins. This watercourse stands out due to its extensive drainage area of 767,000 km^2^, and hosts the two largest iron ore deposits globally: the Carajás mine in Pará State and the Serra do Carmo iron deposit in Tocantins State^19^. Situated in the transitional area between the Amazon and the Cerrado biomes, it plays a crucial ecological role^20^. The river harbors a highly diverse ichthyofauna, boasting approximately 300 species, 126 genera, and 34 families, predominantly Characiformes, Siluriformes, and Cichlids^21^. Notably, the river is vital for the local population's sustenance, supporting fishing for commerce and subsistence, tourism activities such as river beaches, and traditional crafts. However, over the last 40 years, it has experienced significant degradation, primarily due to the expansion of dams, croplands, irrigation, mining, and aquaculture^19^.

The region faces challenges such as deforestation for agriculture, monoculture (e.g., *Eucalyptus*), and sanitation issues driven by urban growth, leading to soil erosion, sedimentation, and the introduction of pollutants^22^. This degradation affects water and sediment quality, impacting ecological health and community well-being. Several polluted streams flow into the study area, causing unsanitary conditions due to the direct dumping of household waste from bathrooms and kitchens, effectively turning them into open sewers^23^. Elevated levels of metals such as Al, Cu, Fe, Mg, and Se exceeding national standards have been observed, indicating these elements are carried into the Tocantins River as suspended particulate matter^24^. A recent study also found that levels of Al, Cu, Fe, Mg, and Se exceed legal standards. Particularly in urban areas, increased levels of conductivity, total dissolved solids, chlorophyll, NH_4_^+^, and NO_2_ raise concerns about drinking water quality^22^. This is critical for residents who rely on untreated river water for recreational and domestic purposes.

The field study aims to address identified research gaps by providing valuable information on the concentration, spatial distribution, pollution levels, and source apportionment of toxic and essential elements in sediment samples from sampling sites distributed along the middle Tocantins River. Additionally, contamination levels have been assessed using various indices such as the Geoaccumulation Index (Igeo), Contamination Factor (CF), Enrichment Factor (EF), Pollution Load Index (PLI), Sediment Pollution Index (SPI), Potential Ecological Risk Coefficients (Er), and their Integrated Risk Index (RI). The lack of studies and the environmental fragility of the middle Tocantins River highlight the urgent need for greater attention to environmental monitoring of this important water resource. Given that anthropogenic activities, particularly urbanization and deforestation, degrade environmental quality, they lead to compromised water and sediment conditions, posing risks to ecosystems and human health.

## Material and methods

### Study área, sampling, and ethical aspects

**Figure 1:**
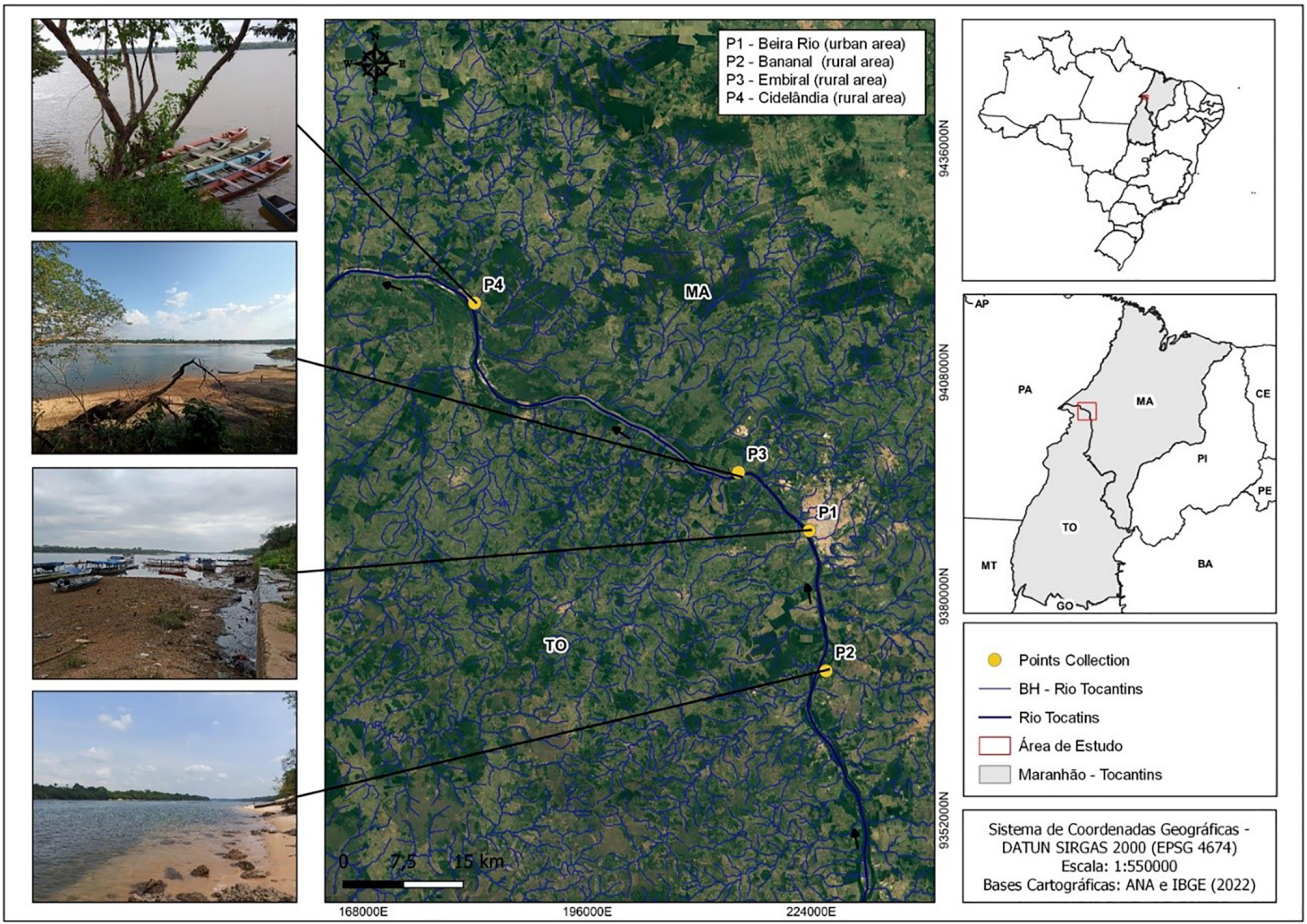
Geographic coordinates of sampling points in the middle Tocantins River, Maranhão, Brazil. P1: Beira Rio (urban area), P2: Bananal (rural area), P3: Embiral (rural area), P4: Cidelândia (rural area).

The study was carried out in the middle Tocantins River within the Imperatriz area of influence (Maranhão, Brazil). There are four sampling station areas, each equipped with GPS georeferencing using the GARMIN 4215 (Fig. 1). This region is strategically vital as a major transportation hub and gateway to the Amazon, often referred to as the portal to the Amazon. Its strong agribusiness and industrial sectors drive economic growth, generating employment and promoting development. The rainiest/coldest period (January–June) and the hot/dry season (July–December) are highlighted^25^.

It’s worth noting that the region faces several challenges, including deforestation on hillsides, intensive exploitation of fishery resources, sedimentation, sanitation issues, and direct disposal of waste and sewage into the water body, all contributing to contamination of the river waters. Consequently, these activities lead to damage to the health of the Tocantins River ecosystem, affecting both fauna and flora and negatively impacting the quality of life for the population relying on the river.

A recent study provides insights into characterizing the region, where P1 is situated in an urbanized area with significant human activity^22^. This site is potentially contaminated due to high vessel traffic, recreational beach use, waste disposal, residues from commercial iceboxes, and urban sewage. On the contrary, P2 has a lower potential for contamination, being situated farther away from the city. P3 is located upstream of a paper and cellulose manufacturing and processing industry, while P4 is approximately 100 km downstream from the city, downstream from the paper and cellulose industry.

The degree of inorganic contamination was monitored from January to December 2023, by collecting sediment samples from sections of the middle Tocantins River. Sediment samples were systematically collected monthly, with three replicates at each designated point, resulting in a total of 12 containers. These samples were carefully preserved in pre-cleaned plastic vials, thoroughly rinsed with metal-free water, and maintained within a cold chain (refrigerated at 4 °C) until they reached the laboratory at the State University of the Tocantina Region of Maranhão (UEMASUL).

Both the field collection and subsequent laboratory procedures followed ethical guidelines and were formally reviewed and approved by the Institutional Ethics Committee of the State University of the Tocantin Region of Maranhão (CEUA No: 1025220722) and the State University of Maranhão (CEUA No 040/2023). Furthermore, scientific activities were conducted with explicit authorization (SISBIO Number: 87310–1) by Normative Instruction No. 748/2022 as outlined in the ICMBio Ordinance.

### Determination of potentially toxic and essential elements in sediments

Samples of 500 g of surface sediments were collected, with subsamples taken from the central part to avoid contamination. Subsequently, the samples were dried in a drying oven at 70˚C (CienlaB) for three days or until a constant mass was obtained. For the determination of total concentrations of elements in sediments, a 0.5-g sample (dry weight) was taken and 1 mL of HCl (50%), 1 mL of nitric acid (50%), and 8 mL of water were added; following the analytical methodology of the U.S. Environmental Protection Agency (US EPA Method 3050B). The digestion was then accelerated in a digestion block (ECO 16, VELP Scientifica) for 2 h at 120ºC.

The concentrations of both potentially toxic elements (aluminium—Al, antimony—Sb, arsenic—As, barium—Ba, cadmium—Cd, chromium—Cr, mercury—Hg, lead—Pb, lithium—Li, nickel—Ni, strontium—Sr, titanium—Ti, and silver—Ag) and essential elements (beryllium—Be, boron—B, selenium—Se, silica—Si, phosphorus—P, copper—Cu, iron—Fe, calcium—Ca, cerium—Ce, potassium—K, magnesium—Mg, manganese—Mn, molybdenum—Mo, sodium—Na, vanadium—V, cobalt—Co, tin—Sn, and zinc—Zn) were determined using inductively coupled plasma emission spectrometry (ICP-EAS) (SHIMADZU, ICPE-9000, Kyoto, Japan). Mercury (Hg) was determined following the specific methodology EPA 245.7:2005^26^.

The results were compared with the National Environmental Council resolution no 454/2012^27^. Additionally, the results were compared with international sediment guidelines, including those from the Canadian Council of Ministers of the Environment (CCME)^28^, the National Oceanic and Atmospheric Administration (NOAA)^29^, and the United States Environmental Protection Agency guidelines for sediments (USEPA)^30^.

### Assessment of anthropogenic pollution in sediments

#### Geoaccumulation index

The Geo-accumulation Index (Igeo) has been calculated for the six studied metals: Al, Cr, Cu, Ni, Pb, and Zn. Igeo serves as a measure to assess metal contamination in sediments, soils, or other environmental samples, providing insights into the degree of accumulation or contamination relative to natural background levels^31^. The data should be processed using Eq. (1):

1 $${\text{Igeo}} = \log_{2} \left( {\frac{{{\text{Cn}}}}{{1.5 \times {\text{Bn}}}}} \right)$$

In this context, Cn represents the measured concentration of the metal in the sediment samples, while Bn denotes the geochemical background value in the Earth’s crust^32^. To address potential variations in background values resulting from lithogenic variations, a factor of 1.5 is introduced^33^.

Muller^31^ categorized the geoaccumulation index into six classes. The classification of contamination levels in sediment, as indicated by the Geo-accumulation Index (Igeo), is determined based on specific ranges^34,35^. When Igeo is less than or equal to 0, it suggests no contamination. For values between 0 and 1, the sediment is categorized as experiencing no contamination to being moderately contaminated. A range of 1–2 indicates a state of moderate contamination, while 2–3 signifies a condition of moderate to heavy contamination. The levels intensify as Igeo progresses: 3–4 suggests heavy contamination, 4–5 indicates contamination from heavy to extremely contaminated, and an Igeo equal to or greater than 5 denotes an extremely contaminated state.

The logarithm to the base 2 is utilized to express the concentration relationship more clearly. It is important to note that understanding the nature of concentration values, especially about background concentration levels, is crucial for interpreting the index correctly. This classification system offers a comprehensive understanding of the contamination degree in sediment samples, thereby assisting in environmental assessments and the development of management strategies.

#### Enrichment factor (EF)

In the present study, the concentrations of elements in the Earth’s crust were utilized as reference values, sourced from Turekian and Wedepohl^36^. The Enrichment Factor (EF) assesses the ratio of elemental concentrations in a given sample to those in the Earth’s upper continental crust (background). This ratio is calculated using Eq. (2) as proposed by Loring^37^. The EF serves as a valuable metric for understanding the extent to which elemental concentrations in a sample deviate from the natural background levels, providing insights into potential anthropogenic influences and environmental impacts.

2 $${\text{EF = }}\frac{{\left( {\frac{{{\text{CM}}}}{{{\text{CX}}}}} \right){\text{sample}}}}{{\left( {\frac{{{\text{CM}}}}{{{\text{CX}}}}} \right){\text{earth crust}}}}$$

CM represents the concentration of the target element in the sample, while CX denotes the concentration of the same element in the Earth's crust. A value of EF greater than 1 indicates enrichment, signifying an elevated concentration relative to the Earth’s crust. Conversely, an EF less than 1 indicates depletion, suggesting a lower concentration compared to the typical Earth's crust. EF is extensively employed in geochemistry and environmental studies to evaluate contamination or the anomalous presence of elements in a sample. Its application provides valuable insights into potential anthropogenic influences and environmental implications.

#### Pollution load index (PLI)

The Pollution Load Index (PLI), developed by Tomlinson et al.^38^, serves as a tool to assess the extent of sediment contamination by metals in diverse locations. This index can be calculated using Eq. (3):

3 $${\text{PLI = }}\sqrt[{\text{n}}]{{{\text{(CF1 }} \times {\text{ CF2 }} \times \cdots {\text{CFi)}}}}$$

The PLI indicates how many times the metal content in the sediment exceeds background levels (Soliman et al. 2021), where n represents the number of metals studied. Therefore, interpreting the PLI involves comparing the calculated value with established limits or standards. A PLI equal to 1 suggests that the area is within acceptable limits and is not significantly polluted. Values above 1 indicate an increase in the pollution load, suggesting rising levels of contamination. According to Soliman et al. (2021), a PLI < 1 indicates “Unpolluted,” PLI > 1 “Polluted,” and PLI > 5 “Very highly polluted”.

#### Sediment pollution index (SPI)

The Sediment Pollution Index (SPI) serves as a comprehensive multi-metals index for assessing sediment quality in terms of both metal content and toxicity. The methodology for calculating SPI, as proposed by Rubio et al.^39^, is outlined, and it is expressed through Eq. (4):

4 $${\text{SPI = }}\frac{{\sum {\text{(EFm }}\; \times {\text{Wm)}}}}{{\sum {\text{Wm}}}}$$

Toxicity weights (Wm) are assigned to each metal, with values of 2, 5, 5, 5, 1, and 1 for Cr, Cu, Ni, Pb, Al, and Zn, respectively^40^^,^^41^. Additionally, EFm represents the Enrichment Factor for each metal. The SPI index is classified into five categories based on the classification by Soliman et al. (2021): 0–2 (Natural sediment), 2–5 (Low-polluted sediment), 5–10 (Moderately polluted sediment), 10–20 (Highly polluted sediment), and > 20 (Dangerously polluted sediment).

#### The potential ecological risk coefficients (Epr) and integrated risk index (RI)

To assess potential toxic hazards posed by metals, the potential ecological risk index (RI) derived by Hakanson^40^ was estimated. According to this index, the potential ecological risk coefficient ($E_{r}^{i}$) for a specific metal and the overall potential ecological risk index (RI) induced by the combined impacts of the studied metals were evaluated using the following formulas:

5 $$C_{f}^{i} = \frac{{C_{s}^{i} }}{{C_{n}^{i} }}$$

6 $$E_{r}^{i} = { }T_{r}^{i}\,x\,C_{f}^{i}$$

7 $${\text{RI}} = \mathop \sum \limits_{i = 1}^{n} E_{r}^{i}$$

The ecological risk indices (RI) and potential ecological risks ($E_{r}^{p}$) were computed using pollution coefficients ($C_{f}^{i}$), metal content in sediment ($C_{s}^{i}$), baseline values ($C_{n}^{i}$), and toxicity coefficients ($T_{r}^{i}$) (Okbah et al. 2018; Soliman et al. 2019). The pollution coefficients for Cr, Cu, Ni, Pb, Al, and Zn were 2, 5, 5, 5, 1, and 1, respectively. Based on the calculated indices, the ecological risk levels were categorized.

RI values below 150 indicate low risk, while values between 150 and 300 suggest moderate risk^42^. RI values ranging from 300 to 600 indicate considerable risk, and values equal to or exceeding 600 represent significant risk. Similarly, Epr values below 40 signify low risk, while values between 40 and 80 indicate moderate risk. Considerable risk is denoted by Epr values between 80 and 160, while high risk corresponds to values between 160 and 320. Epr values equal to or exceeding 320 indicate very high risk. These categorizations enable a comprehensive assessment of ecological risk levels based on metal concentrations in sediment samples.

### Statistical analysis

To evaluate significant differences in the average total concentration of essential and toxic elements across sampling stations or within the same station during different periods of 2023, Analysis of Variance (ANOVA) was employed. Before conducting the analysis, it was essential to perform the Kolmogorov–Smirnov normality test and Levene’s test to assess variance homogeneity. The Tukey test (*p* < 0.05) was also employed to compare means. The data were analyzed using the statistical software SPSS version 22.

To identify the association between potentially toxic and essential elements analyses, statistical tools such as correlation matrix and principal component analysis (PCA) were explored on raw data using the free software PAST 4.03 (latest version 2020). The technique reduces data dimensionality, emphasizing principal components explaining most variability, and aiding interpretation of inter-variable relationships for a comprehensive sediment quality analysis.

## Results and discussion

### Spatial distribution and annual average concentration of potentially toxic and essential elements in sediments from the middle Tocantins River

**Figure 2:**
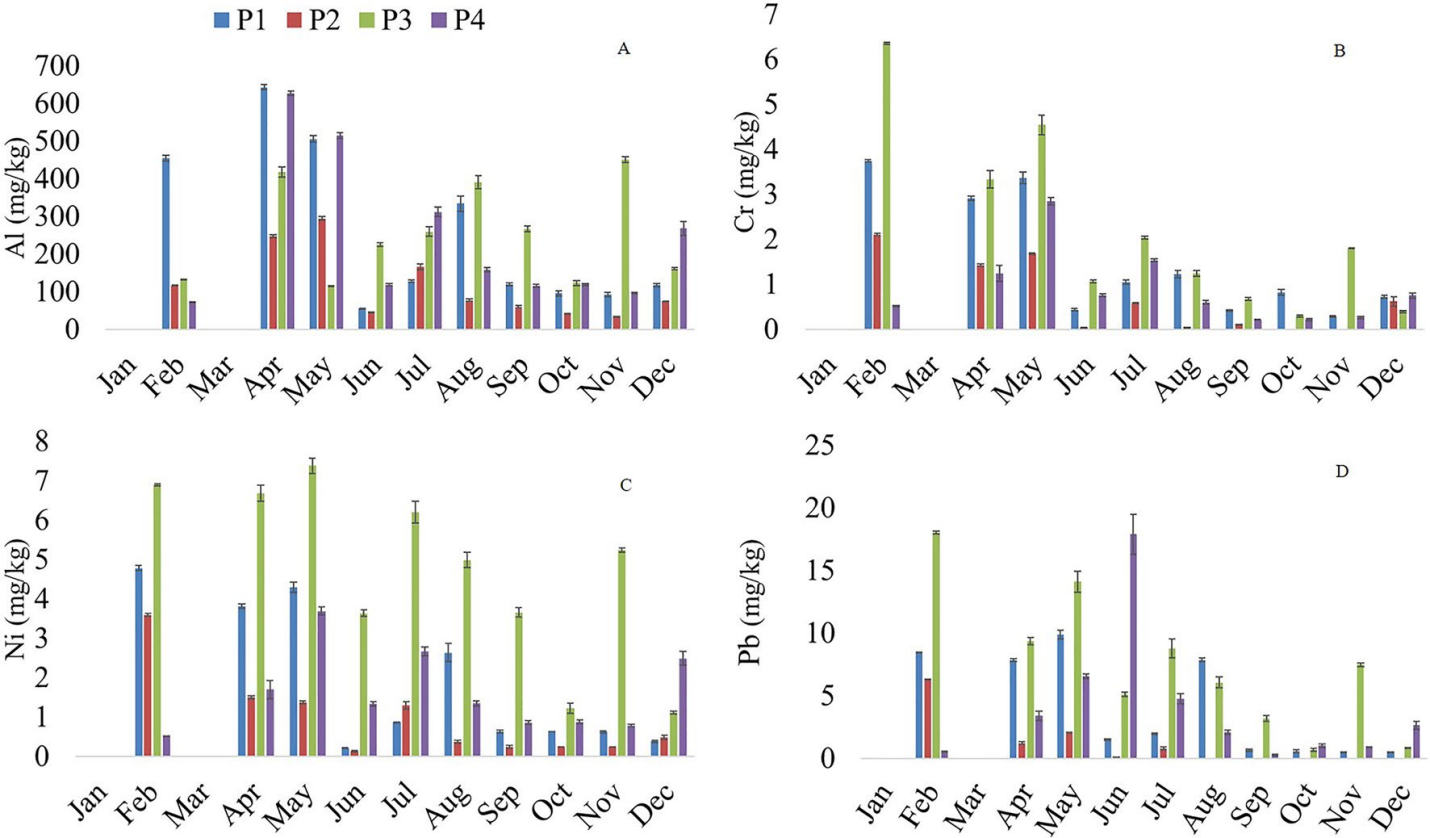
Spatial distribution of potentially toxic elements in the sediments of the middle Tocantins River throughout 2023, Maranhão, Brazil. P1: Beira Rio (urban area), P2: Bananal (rural area), P3: Embiral (rural area), P4: Cidelândia (rural area). Rainiest/coldest period (January to June) and the hot/dry season (July to December) (Koppen and Geiger, 1928). A: Aluminium (Al); B: Chromium (Cr); C: Nickel (Ni); D: Lead (Pb). Values represent mean concentrations (mg/kg) of each metal. Error bars indicate the standard deviation (SD) of the mean concentrations.

The sediment monitoring of the middle Tocantins River has revealed significant trends in the concentration of chemical elements throughout the year 2023. Notably, the months of February, April, and May show heightened levels of Al (Fig. 2A), Cr (Fig. 2B), Ni (Fig. 2C), and Pb (Fig. 2D). This period coincides with the rainiest and coldest months (January–June). However, these elements are undetectable (< 0.01 mg/kg) in January and March due to excessive rainfall, which dilutes the metals.

The rains began in January weakly; February saw heavy rain and thunderstorms punctuated by sunny days, while March brought continuous and heavy rainfall^43^. This seasonal fluctuation suggests a multifaceted interaction influenced by climatic and seasonal factors. At Site P1, there is a higher presence of Al, while Ni exhibits a greater concentration in P3 (Fig. 2). Silva et al.^44^ state that urban activities disrupt natural metal levels in sediments and aquatic ecosystems by altering geochemical processes that mobilize, transport, and disperse hazardous metals. Pb seems to be more prevalent in P3, except in June, where a greater concentration is observed at point P4. These concentration fluctuations underscore the necessity for a more thorough investigation to comprehend the temporal patterns and potential influencing factors linked to the presence of these elements in the waters of the middle Tocantins River. Additionally, spatial analysis of sediments can provide insights into the transport and deposition of contaminants^15^.

Senze et al.^45^ also observed that the seasonal variations were reflected in the levels of Al detected in the sediments. The authors noted that across all rivers and their tributaries, autumn values significantly exceeded spring levels, indicating aluminum retention in sediments during the summer months. Conversely, in water, the opposite trend was observed, likely attributed to abundant precipitation and the leaching of aluminum from the catchment. Acioly et al.^22^ reported that February, part of the rainiest and coldest period (January–July), recorded the highest values of elements in the water of the middle Tocantins River. In this recent study, annual concentrations of Al exceeded both national and international water quality standards (P1: 0.69 mg/L,P2: 0.71 mg/L,P3: 0.63 mg/L; P4: 0.58 mg/L). Elevated levels of essential elements such as Cu, Fe, Mn, and Se also raise significant concerns for environmental and health quality.

Aluminum demonstrated higher values among the potentially toxic elements; however, it is noteworthy that national and international standards do not provide specific limits for this element (Table 1). Meanwhile, values for Cr, Ni, and Pb exceeded only the sediment guideline set by the National Oceanic and Atmospheric Administration^29^. The same holds for essential elements,Cu and Zn surpassed the guidelines established by NOAA. No statistical differences were observed in the average concentrations among the studied sections of the middle Tocantins River.

**Table 1:** Comparison of the concentration of potentially toxic and essential elements (annual average, mg/kg) in sediment from the middle Tocantins River with national and international regulations.

Elements	CONAMA	CCME	USEPA (Not Polluted)	NOAA		Tocantins River			
				TEL	PEL	P1	P2	P3	P4
Potentially toxic elements									
Al						254.97 ± 43.13	116.04 ± 16.94	254.51 ± 29.74	240.32 ± 39.91
As	5.9	5.9	3			0.57 ± 0.13	0.15 ± 0.06	2.38 ± 0.34	0.63 ± 0.13
Au						3.08 ± 0.34	1.52 ± 0.20	5.29 ± 0.60	2.75 ± 0.27
Ba						5.10 ± 0.83	2.66 ± 0.31	9.64 ± 1.30	4.35 ± 0.48
Cr	37.3	37.3	25	0.0523	0.16	1.50 ± 0.25*	0.66 ± 0.15*	2.18 ± 0.39*	0.90 ± 0.18*
Hg	0.17	0.17		0.13	0.7	< 0.06	< 0.06	< 0.06	< 0.06
In						1.86 ± 0.31	1.08 ± 0.26	3.51 ± 0.54	1.81 ± 0.25
Ni	18		20	0.0159	0.0428	1.78 ± 0.36*	0.90 ± 0.21*	4.70 ± 0.54*	1.63 ± 0.25*
Pb	35	35	40	0.03024	0.112	3.98 ± 0.73*	1.05 ± 0.35*	7.37 ± 1.19*	4.02 ± 1.60*
Sb						1.41 ± 0.28	0.62 ± 0.17	4.04 ± 0.51	1.55 ± 0.22
Essential elements									
B						2.05 ± 0.43	1.14 ± 0.24	2.25 ± 0.34	1.07 ± 0.18
Ca						291.65 ± 26.34	133.19 ± 17.19	399.93 ± 48.36	145.47 ± 18.29
Cu	35.8	35.7	25	0.0187	0.39	1.32 ± 0.13*	0.60 ± 0.05*	2.11 ± 0.23*	0.94 ± 0.10*
Co						2.98 ± 0.43	1.18 ± 0.24	5.35 ± 0.66	2.58 ± 0.34
Fe						774.50 ± 139.39	378.81 ± 57.50	1105.75 ± 163.97	549.99 ± 83.79
K						16.72 ± 3.82	11.75 ± 2.76	31.92 ± 6.47	14.87 ± 2.33
P						2.72 ± 0.43	0.79 ± 0.20	5.29 ± 0.72	1.85 ± 0.32
Mg						47.19 ± 8.02	30.00 ± 4.50	136.02 ± 17.60	50.35 ± 6.77
Mn						13.63 ± 1.42	7.95 ± 0.96	29.14 ± 2.69	14.04 ± 1.49
Na						15.88 ± 3.09	11.69 ± 1.48	15.02 ± 2.38	11.38 ± 1.65
S						9.24 ± 2.77	12.09 ± 7.34	24.74 ± 4.70	4.32 ± 0.66
Se						7.50 ± 0.93	3.55 ± 0.67	13.37 ± 1.55	6.16 ± 0.70
Si						255.84 ± 31.71	186.83 ± 20.04	341.25 ± 46.73	218.79 ± 22.74
Sn						3.85 ± 0.54	2.27 ± 0.44	8.55 ± 0.99	3.52 ± 0.41
V						1.57 ± 0.24	1.01 ± 0.26	2.12 ± 0.32	1.11 ± 0.18
Zn	123	123		0.124	0.271	4.15 ± 1.11*	2.11 ± 1.04*	9.03 ± 4.35*	3.08 ± 1.58*

P1: Beira Rio (urban area), P2: Bananal (rural area), P3: Embiral (rural area), P4: Cidelândia (rural area). CONAMA: National Environmental Council resolution no 454/2012^27^. USEPA: United States Environmental Protection Agency guidelines for sediments^30^. CCME: Canadian Council of Ministers of the Environment^28^. NOAA: National Oceanic and Atmospheric Administration^29^, Sediment Guidelines. TEL: Threshold Effects Level. PEL: Probable Effects Level. These are metrics similar to the effects range metrics (i.e., Effects Range Low and Effects Range Median) used to assess the potential effects of contaminants on sediments. The Threshold Effects Level is the average of the 50th percentile and the 15th percentile of a dataset and the Probable Effects Level is the average of the 50th percentile and the 85th percentile of a dataset. *Parameter concentrations above the quality standards of some regulations in comparison. The Tukey test was performed (*p* < 0.05) for mean comparison,where “a” and “b” alone denote significant differences about other groups. The detection limit was 0.01 mg/kg for Al, Au, Hg, Ca, Pb, S, and Si,0.02 mg/kg for Ar, B, Ba, Cu, Cr, Fe, K, Mg, Mn, Na, Ni, Se, Sn, and Zn,and 0.03 mg/kg for In, Cd, Co, P, Sb, and V.

The elements with the highest mean values followed this order: Fe > Al > Si > Mg > K > Na > S. The sediment’s mercury content was below the detection limit (Table 1). Manganese and Iron are not explicitly addressed in national and international standards. However, according to Aiman et al.^46^, its safety level is established at 30 mg/kg for sediment quality guidelines (SGV). The same source mentions a safety level of 15 mg/kg for Fe. It is crucial to underscore the lack of specific regulations for aluminum, highlighting a potential gap in standards. Continued monitoring is essential to assess the environmental and health implications of exceeding guidelines for other elements.

Aluminum ranks as the third most abundant element and the predominant metal in the Earth’s crust^47^. While commonly present in rocks where it is considered inert and non-bioavailable, weathering of these minerals gradually releases potentially toxic forms of aluminum into the environment, such as Al_3_^+^ and Al hydroxides^47,48^. Both natural and human activities contribute to the presence of aluminum in aquatic systems. In sediment, aluminum is typically inert when bound with dissolved organic carbon (DOC) or in the form of silt or clay, serving as a storage reservoir. However, changing conditions, such as a decrease in pH, can mobilize aluminum back into the water column^49,50^. In such cases, riverbed sediment acts as a pollution store. Health effects of aluminum exposure include impacts on the respiratory, nervous, skeletal, hematological, cardiovascular, hepatic, renal, and endocrine systems, as well as skin, eyes, and body weight^51–53^.

According to Ohiagu et al.^54^, elements such as Cr, As, Pb, Cu, Fe, Cd, Zn, and Ni are among the metals raising high public health concerns. Several studies indicate that the presence of Cr, Cd, Pb, Mn, and Ni above threshold limits suggests potential carcinogenic and genotoxic effects on humans^55^,Balali-Mood et al.^56^. This situation is alarming and demands continuous monitoring, public awareness, and stringent governmental policy. Notably, Point P3 exhibited slightly elevated values for Cr (2.18 mg/kg), Ni (4.70 mg/kg), and Pb (7.37 mg/kg).

3.2. Assessment of anthropogenic pollution in sediments: Geoaccumulation Index, Enrichment Factor, Contamination Factor, Pollution Load Index, Sediment Pollution Index, and Potential Ecological Risk Index.

For the Igeo, the samples of Al, Cr, and Cu consistently show only negative values, indicating a low level of pollution (Table 2). Among the metals analyzed, Ni and Pb stand out as the highest contaminants. When examining the average data for 2023, only Pb recorded a positive value for P3 (0.54); nevertheless, it still falls within the range of no contamination to moderately contaminated. However, considering the maximum values obtained for metal concentration in the Igeo equation, both Ni and Pb recorded values higher than 1 for points P1 (1.50), P3 (1.92), and P4 (3.00).

**Table 2:** Geoaccumulation index and enrichment factor for investigated metals in the sediment from the Middle Tocantins River, Maranhão, Brazil.

Elements	Index value	Igeo				Enrichment factor			
		P1	P2	P3	P4	P1	P2	P3	P4
Al	Min	− 3.96	− 4.83	− 3.52	− 3.47	0.00062	0.00028	0.00083	0.00088
	Average	− 2.39	− 3.26	− 2.40	− 2.45	0.00319	0.00145	0.00318	0.00300
	Máx	− 2.82	− 2.82	− 1.61	− 1.39	0.01097	0.00433	0.00858	0.01053
Cr	Min	− 4.29	− 4.80	− 3.91	− 4.48	0.00078	0.00056	0.00189	0.00067
	Average	− 2.00	− 2.88	− 1.72	− 2.42	0.01667	0.00733	0.02422	0.01000
	Máx	− 1.20	− 2.11	− 0.95	− 1.47	0.05278	0.02667	0.07844	0.04078
Cu	Min	− 2.18	− 3.04	− 2.08	− 2.63	0.01133	0.00489	0.01333	0.00667
	Average	− 1.49	− 2.35	− 1.08	− 1.93	0.02933	0.01333	0.04711	0.02089
	Máx	− 0.84	− 1.72	− 0.37	− 1.14	0.08356	0.02800	0.13644	0.06467
Ni	Min	− 5.15	− 7.14	− 2.20	− 4.05	0.00138	0.00016	0.01926	0.00441
	Average	− 1.16	− 2.10	− 0.43	− 1.32	0.02618	0.01324	0.06912	0.02397
	Máx	0.61	− 0.10	1.08	0.02	0.08588	0.05882	0.17471	0.06941
Pb	Min	− 2.39	− 2.85	− 1.79	− 1.73	0.035	0.022	0.0545	0.0595
	Average	− 0.22	− 2.12	0.54	− 0.03	0.199	0.0525	0.369	0.201
	Máx	1.50	0.85	1.92	3.00	0.695	0.33	0.93	2.41
Zn	Min	− 6.01	− 7.62	− 3.34	− 6.01	0.00105	0.0002526	0.01036	0.0003368
	Average	− 1.38	− 2.53	− 0.65	− 2.06	0.04368	0.02221	0.09474	0.03242
	Máx	− 0.54	− 0.41	0.92	− 0.14	0.2653	0.3021	1.347	0.4811

Min: minimum value observed for the element; Average: average value observed for the element; Máx: maximum value observed for the element. P1: Beira Rio (urban area), P2: Bananal (rural area), P3: Embiral (rural area), P4: Cidelândia (rural area). Igeo values: ≤ 0 (no contamination), 0–1 (none to moderate contamination), 1–2 (moderate contamination), 2–3 (moderate to heavy contamination), 3–4 (heavy contamination), 4–5 (heavy to extreme contamination), ≥ 5 (extreme contamination). An EF > 1 indicates enrichment (elevated concentration relative to Earth's crust), while an EF < 1 indicates depletion (lower concentration than typical Earth's crust).

The calculations indicate a minimal presence of zinc pollution in the samples. The Igeo serves as a valuable tool for evaluating the prevailing contamination levels in specific sedimentary areas^57,58^. For the majority of points and situations, the Igeo values were predominantly negative, indicating a lack of contamination. These values offer a descriptive investigation of sediment contamination status and the accumulation profile of heavy metals^59,60^. The average EF for the studied metals exhibited a decrease in the following order: Pb > Ni > Zn > Cu > Cr > Al. Moreover, when evaluating the maximum metal concentration values in the EF equation, Pb was the sole element surpassing a value of 1 (P4 = 2.41), whereas Zn recorded a value above 1 for P3 (P3 = 1.347) (Table 2). The EF stands out as a crucial index for identifying the sources of metals in both soil and sediment^61^. A value of EF greater than 1 indicates enrichment, signifying an elevated concentration relative to the Earth’s crust^62,63^. Conversely, an EF less than 1 indicates depletion, suggesting a lower concentration compared to the typical Earth’s crust.

The mean values for the studied metals revealed a higher PLI for Ni (0.235) and Pb (0.184), both observed at P3 (Table 3). On the other hand, considering the observed maximum values obtained through ICPE analysis, the PLI hierarchy is as follows: Pb (1.205 at P4) > Zn (1.041 at P3) > Ni (0.595 at P3) > Cr (0.282 at P3) > Cu (0.246 at P3). These results suggest that, based on the mean values, there is no imminent concern. However, at certain moments or periods of the year, there is an escalation in pollution levels (PLI > 1 “Polluted” according to Soliman et al. 2021), particularly for lead and zinc. This emphasizes the importance of monitoring and implementing environmental management strategies to mitigate further contamination risks.

**Table 3:** Pollution load index and sediment pollution index for investigated metals in the sediment from the middle Tocantins River, Maranhão, Brazil.

Elements	Index value	Pollution load index (PLI)				Global	Sediment pollution index (SPI)				Global
		P1	P2	P3	P4		P1	P2	P3	P4	
Al	Min	0.089	0.074	0.112	0.097	0.135	0.00062	0.00028	0.00083	0.00088	0.011
	Average	0.153	0.114	0.147	0.145		0.00319	0.00145	0.00318	0.00300	
	Máx	0.200	0.151	0.182	0.196		0.01097	0.00433	0.00858	0.01053	
Cr	Min	0.003	0.002	0.007	0.002	0	0.00156	0.00112	0.00378	0.00134	0.044
	Average	0.060	0.026	0.087	0.036		0.03334	0.01466	0.04844	0.020	
	Máx	0.190	0.096	0.282	0.147		0.10556	0.05334	0.15688	0.08156	
Cu	Min	0.020	0.009	0.024	0.012	0.066	0.01133	0.00489	0.01333	0.00667	0.043
	Average	0.053	0.024	0.084	0.038		0.02933	0.01333	0.04689	0.02089	
	Máx	0.150	0.050	0.246	0.116		0.08356	0.028	0.13644	0.06467	
Ni	Min	0.005	0.0005	0.065	0.015	0.128	0.00138	0.00016	0.01926	0.00441	0.024
	Average	0.089	0.045	0.235	0.081		0.01309	0.00647	0.03397	0.01176	
	Máx	0.292	0.200	0.595	0.236		0.04294	0.02941	0.06985	0.02765	
Pb	Min	0.017	0.011	0.027	0.030	0.305	0.035	0.022	0.0545	0.0595	0.444
	Average	0.099	0.026	0.184	0.100		0.199	0.052	0.368	0.201	
	Máx	0.347	0.165	0.465	1.205		0.695	0.330	0.930	2.410	
Zn	Min	0.0008	0.00019	0.008	0.00026	0.112	0.00105	0.00025	0.01037	0.000337	0.090
	Average	0.034	0.017	0.073	0.025		0.01379	0.00705	0.03063	0.01047	
	Máx	0.205	0.234	1.041	0.372		0.08526	0.09726	0.43158	0.15410	

Min: minimum value observed for the element; Average: average value observed for the element; Máx: maximum value observed for the element. P1: Beira Rio (urban area), P2: Bananal (rural area), P3: Embiral (rural area), P4: Cidelândia (rural area). A PLI < 1 indicates “Unpolluted,” PLI > 1 “Polluted,” and PLI > 5 “Very highly polluted.” SPI index: 0–2 (Natural), 2–5 (Low), 5–10 (Moderate), 10–20 (High), > 20 (Dangerous).

A variety of pollution indices are at our disposal for evaluating the quality of soil and sediment. The selection of these indices is intricately tied to specific objectives, taking into account factors such as contamination levels, metal origins, and potential ecological risks^64–66^. Furthermore, the mathematical and statistical methodology behind these indices makes them practical and highly efficient tools for environmental studies^61^. Each index specializes in investigating contamination, emphasizing the need to tailor the choice based on the distinct goals of the assessment.

Similar to the PLI index, the SPI provides a composite measure of sediment contamination by various pollutants, allowing for a comprehensive assessment of sediment quality concerning pollution^67,68^. In this study, all investigated metals showed low pollution levels (Natural sediment), with Pb being the most concerning, having a global SPI value of 0.444 (Table 3). The overall PLI value was also higher for Pb. However, when considering the maximum concentration of metals found, Pb stands out with a SPI of 2.410 (P4), classified as “Low-polluted” sediment. Despite Al showing among the highest mean values, its accumulation index values were low, likely due to its high reference value (a relatively abundant element in the Earth’s crust).

The estimation results of potential ecological risk coefficients (Epr) and their integrated risk index (RI) for sediment samples are presented in Table 4. The order of Epr for the studied metals is Pb > Ni > Zn > Cu > Cr > Al. Consequently, the estimated Er values for these elements are all lower than 40, indicating a low level of ecological risk^42^. As for the RI values, the combined potential ecological risks are assessed to be at a low degree of contamination for the majority of the studied sites. The highest RI value was observed for Pb (15.749) (Table 4). However, even with this value, the overall risk level for the middle Tocantins River remains classified as “Low-grade” in terms of potential ecological risk.

**Table 4:** The potential ecological risk index for investigated metals in the sediment from the middle Tocantins River, Maranhão, Brazil.

Sampling site	Index value	Potential ecological risk coefficients (Epr)					
		Al	Cr	Cu	Ni	Pb	Zn
P1	Min	0.001	0.016	0.056	0.070	0.175	0.001
	Average	0.003	0.033	0.147	0.131	0.995	0.044
	Máx	0.008	0.106	0.419	0.416	1.724	0.265
P2	Min	0.001	0.011	0.024	0.009	0.110	0.000
	Average	0.002	0.015	0.067	0.059	0.262	0.022
	Máx	0.004	0.053	0.140	0.294	0.825	0.303
P3	Min	0.001	0.038	0.067	0.101	0.272	0.010
	Average	0.003	0.052	0.233	0.410	1.842	0.096
	Máx	0.006	0.157	0.687	0.882	2.790	1.347
P4	Min	0.001	0.013	0.033	0.022	0.297	0.000
	Average	0.003	0.020	0.104	0.114	0.402	0.033
	Máx	0.008	0.082	0.305	0.319	6.055	0.482
Integrated risk index (RI)		0.041	0.596	2.282	2.827	15.749	2.603

P1: Beira Rio (urban area), P2: Bananal (rural area), P3: Embiral (rural area), P4: Cidelândia (rural area). Min: minimum value observed for the element; Average: average value observed for the element; Máx: maximum value observed for the element. Epr values: < 40 (Low risk), 40–80 (Moderate risk), 80–160 (Considerable risk), 160–320 (High risk), ≥ 320 (Very high risk). RI values: < 150 (Low risk), 150–300 (Moderate risk), 300–600 (Considerable risk), ≥ 600 (Significant risk).

All estimations were conducted across the minimum, average, and maximum values observed in sediment samples collected in 2023. This approach allows for a comprehensive understanding of the temporal variability of the risks involved. By considering the full range of values within the sediment samples, we can better grasp how ecological risk levels fluctuate over time. This comprehensive analysis enhances our ability to assess and manage potential environmental risks associated with metal contamination in sediment ecosystems. Comprehensive ecological risk assessment is of great significance for the restoration of watershed ecosystem health, and the appropriate and effective assessment method is the premise of ecological risk assessment^66^.

### Correlation coefficients and principal component analysis (PCA)

The strongest positive correlations are observed within specific pairs of data: Cu/Si (r = 0.999), Ni/Sn (r = 0.999), Au/Co (r = 0.999), As/Mg (r = 0.999), and Cr/Fe (r = 0.996) (Table S1). A negative correlation was not observed. Correlation coefficients offer valuable insights into the strength and direction of relationships among different parameters related to water quality. They are commonly used to analyze associations between persistent contaminants in aquatic ecosystems^69–71^. A coefficient closer to 1 or − 1 indicates a stronger correlation, while values near 0 suggest a weaker or nonexistent linear relationship.

Correlation coefficients provide valuable insights into the strength and direction of relationships among analyzed parameters, enhancing our understanding of sediment quality dynamics and potential aquatic contaminant sources^72,73^. Consequently, numerous studies utilize both univariate and multivariate statistical analyses to identify highly correlated sediment pollutants and relevant industries^74,75^. This enables researchers to effectively trace these pollutants and industries, leading to the development of more efficient pollution management strategies and mitigation efforts.

**Figure 3:**
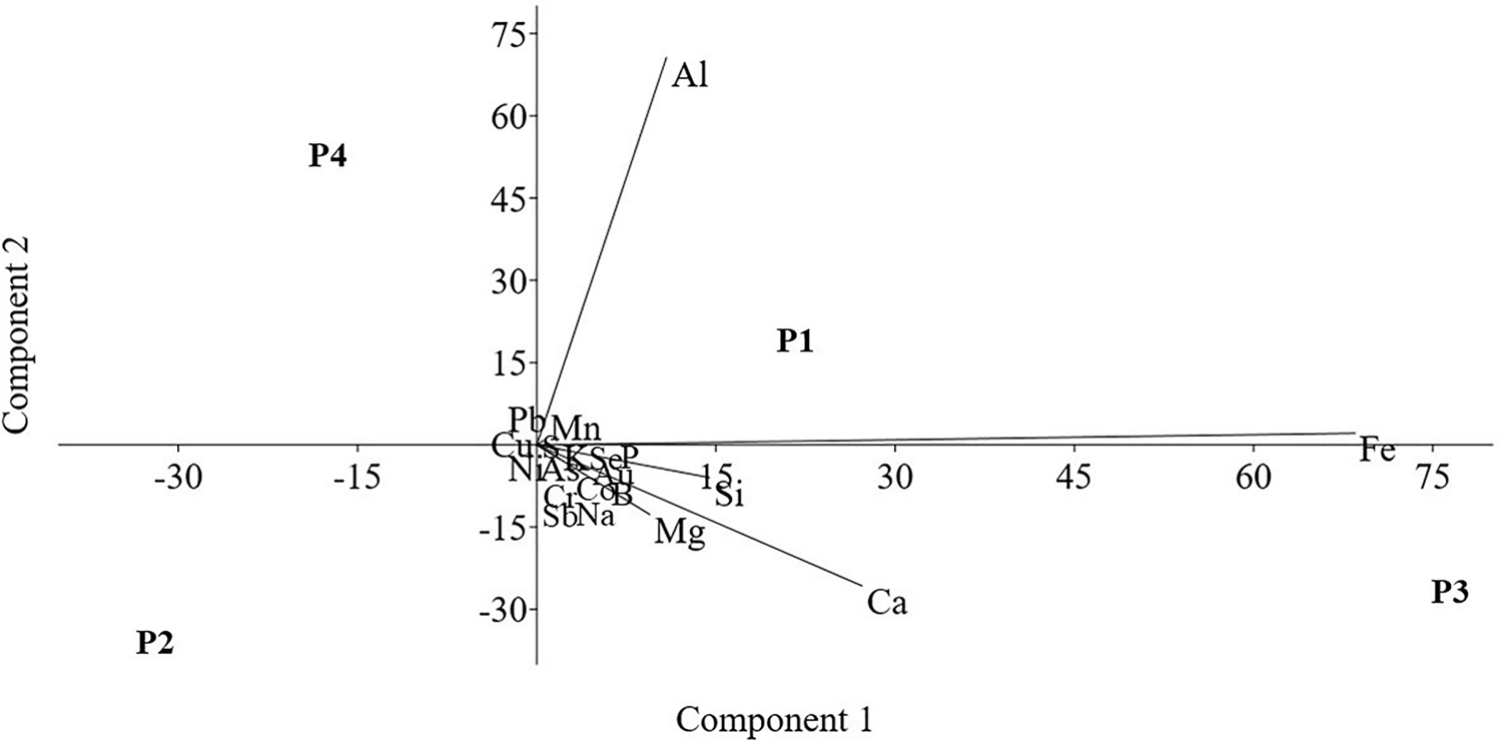
Principal component analysis (PCA) of sediment quality monitoring data of the middle Tocantins River, Maranhão, Brazil. P1: Beira Rio (urban area), P2: Bananal (rural area), P3: Embiral (rural area), P4: Cidelândia (rural area).

The PCA results indicate that the first two components explain 99.38% of the data variability (Fig. 3). The first component accounts for 97.5% of the variance and is positively loaded with As, B, Ba, Ca, Co, Cr, Cu, and Fe. Conversely, the second component accounts for 1.92% of the variance and is positively loaded with Al and Pb, while exhibiting negative loadings for Ca, Mg, S, and Zn. In this study, the correlation matrix was used to better capture the relationships between metals, minimizing the impact of different variance scales among them. The loadings, scores, and eigenvalues of the principal component analysis are available in the supplementary material (Tables S2, S3, and S4).

Highlighting the considerable role of aluminum in sediment contamination, PCA offered valuable insights into the underlying dynamics. These sediments can serve as significant sources of metal contamination in aquatic environments, as deposited or stored contaminants may re-suspend or dissolve back into the water. Hence, employing this technique was crucial for pinpointing pollution sources and extracting reliable information to elucidate clearer relationships^76,77^.

The PCA analysis unveiled three distinct groups based on sediment quality. The first group comprised stations P1 (located in an urban area) and P3 (close to industrial waste discharge). P1 showed a positive association with the first and second components but a negative association with the third, whereas P3 exhibited a positive association with the first and third components. The second group solely included station P2, situated near the village of “Bananal” in a rural area, which displayed a negative association with all PCA components. The third group consisted solely of station P4, also located in rural areas but in proximity to industrial waste discharge (from pulp and paper production). P4 showed a positive association with both the second and third components but a negative association with the first. This underscores the effectiveness of PCA in identifying pollution sources and providing valuable insights for conservation and management efforts.

This research is one of the few studies on the quality of sediments in the middle Tocantins River, highlighting high concentrations of metals such as Al, Cr, Ni, Pb, Cu, and Zn in an urbanized region of the river. Unregulated urbanization has led to environmental changes, adding high loads of untreated domestic and industrial wastewater, and altering biological, physical, and chemical processes in natural systems, directly or indirectly affecting the population’s quality of life^78–80^. The river’s maximum flow is approximately 11.000 m^3^/s, contributing 8.388 t/day of suspended solid discharge to the ocean^81^, supporting marine biodiversity at its mouth. Additionally, the Tocantins River basin is among the most degraded in the Amazon^19,82^. According to Schmitz^83^, tropical deforestation and unsustainable policies pose current challenges in this basin, which serves as the ecotone between the Amazon and Cerrado biomes. This region is a primary target for agricultural expansion in Brazil, as indicated in Presidential Decree 8447 of 2015^19,84^. Furthermore, Brazil stands as the world’s second-largest agricultural producer and is forecasted to undergo substantial growth in agricultural output over the next four decades^85^^,^^86^. Despite these challenges, freshwater ecosystems in this region play vital roles in biodiversity preservation, water purification, and flood control.

In this scenario, this region emerges as a pivotal case study, and measures might be implemented to decrease the levels of these potentially toxic elements. Effective measures to address these issues include the treatment of industrial and urban effluents and the reforestation and conservation of riparian forests^87,88^. Industries and urban areas should be required to properly treat their effluents before discharging them into the river. Water treatment technologies such as chemical precipitation, adsorption, and filtration can effectively remove heavy metals^89–91^. Additionally, vegetation along riverbanks (riparian forests) acts as a natural filter, reducing the amount of sediment and contaminants reaching the river^92,93^. Reforestation projects can help restore these areas, enhancing their capacity to mitigate pollution and protect aquatic ecosystems.

Often, environmental degradation stems from a lack of environmental responsibility and ineffective public policies. Thus, the ongoing deforestation, even in conservation-oriented scenarios, raises questions about the effectiveness of current protection measures, highlighting the need for reassessment and strengthening of conservation strategies. Therefore, ongoing monitoring and enforcement, along with education and awareness efforts, are essential^94–96^. Establishing a continuous monitoring system for pollution levels and rigorous enforcement to ensure adherence to environmental laws is crucial. Furthermore, educational campaigns for local communities and industries on the impacts of pollution and the importance of sustainable practices can foster changes in behavior.

## Conclusion

The levels of aluminum, iron, manganese, and selenium exceeded legal standards through 2023. Although aluminum showed the highest values among the potentially toxic elements, there are no specific limits in national and international standards for this element. Chromium, nickel, copper, zinc, and lead only surpassed the guidelines established by NOAA (National Oceanic and Atmospheric Administration). The seasonal fluctuation indicates a complex dynamic influenced by climatic or seasonal factors. The rainy months of February, April, and May showed heightened levels of aluminum, chromium, nickel, and lead. Particularly, in urban área P1 had a higher presence of Al, while Ni and Pb were more concentrated in P3.

Igeo indicates low pollution levels, with lead and nickel standing out in urban areas (P1) and near the pulp and paper industry (P3 and P4). The EF highlights high concentrations of Pb (P4) and Zn (P3). Both PLI and SPI indices raise concerns regarding Pb (P4) and Zn (P3) concentrations at specific times of the year. The overall risk level for the middle Tocantins River remains classified as “Low-grade” concerning potential ecological risk. These findings stress the importance of continuous monitoring and interventions to uphold water and environmental quality in the region.

### Supplementary Information

Supplementary Tables.

## Data Availability

The datasets used and/or analyzed during the current study are available from the corresponding author on reasonable request.
