# Supplementary material for: Levels of potentially toxic and essential elements in Tocantins River sediment: health risks at Brazil’s Savanna-Amazon interface
Source: Sci Rep. 2024 Aug 4;14:18037. doi: 10.1038/s41598-024-66570-4 (PMC11298526; doi:10.1038/s41598-024-66570-4)
Supplement: Supplementary file 1 — Supplementary Tables. [file 41598_2024_66570_MOESM1_ESM.docx]

**Table S1.** Pearson correlation coefficients for sediment quality monitoring data of the middle Tocantins River, Maranhão, Brazil.

|  | Al | As | Au | B | Ba | Ca | Co | Cr | Cu | Fe | Hg | In | K | Mg | Mn | Na | Ni | P | Pb | S | Sb | Se | Si | Sn | V | Zn |
| --- | --- | --- | --- | --- | --- | --- | --- | --- | --- | --- | --- | --- | --- | --- | --- | --- | --- | --- | --- | --- | --- | --- | --- | --- | --- | --- |
| Al |  | 0.436 | 0.266 | 0.386 | 0.336 | 0.356 | 0.253 | 0.300 | 0.288 | 0.259 | 0.541 | 0.325 | 0.430 | 0.461 | 0.361 | 0.393 | 0.421 | 0.302 | 0.187 | 0.894 | 0.389 | 0.299 | 0.309 | 0.407 | 0.354 | 0.412 |
| As | 0.564 |  | 0.030 | 0.282 | 0.015 | 0.139 | 0.035 | 0.095 | 0.053 | 0.080 | 0.015 | 0.010 | 0.006 | 0.000 | 0.004 | 0.500 | 0.002 | 0.036 | 0.062 | 0.160 | 0.002 | 0.026 | 0.044 | 0.003 | 0.087 | 0.014 |
| Au | 0.734 | 0.970 |  | 0.192 | 0.005 | 0.082 | 0.000 | 0.040 | 0.012 | 0.022 | 0.060 | 0.005 | 0.024 | 0.037 | 0.013 | 0.362 | 0.022 | 0.006 | 0.013 | 0.260 | 0.020 | 0.002 | 0.012 | 0.018 | 0.047 | 0.021 |
| B | 0.615 | 0.718 | 0.808 |  | 0.187 | 0.028 | 0.187 | 0.060 | 0.111 | 0.087 | 0.238 | 0.236 | 0.213 | 0.289 | 0.265 | 0.041 | 0.240 | 0.139 | 0.256 | 0.309 | 0.282 | 0.167 | 0.118 | 0.229 | 0.060 | 0.176 |
| Ba | 0.664 | 0.985 | 0.995 | 0.813 |  | 0.074 | 0.007 | 0.038 | 0.012 | 0.026 | 0.031 | 0.004 | 0.007 | 0.019 | 0.008 | 0.370 | 0.008 | 0.005 | 0.033 | 0.197 | 0.012 | 0.002 | 0.009 | 0.018 | 0.038 | 0.006 |
| Ca | 0.644 | 0.861 | 0.918 | 0.972 | 0.926 |  | 0.080 | 0.008 | 0.031 | 0.022 | 0.113 | 0.108 | 0.091 | 0.144 | 0.127 | 0.131 | 0.109 | 0.045 | 0.138 | 0.216 | 0.139 | 0.063 | 0.033 | 0.101 | 0.006 | 0.067 |
| Co | 0.747 | 0.965 | **0.999** | 0.813 | 0.993 | 0.920 |  | 0.038 | 0.012 | 0.020 | 0.067 | 0.008 | 0.028 | 0.042 | 0.016 | 0.353 | 0.027 | 0.007 | 0.012 | 0.271 | 0.024 | 0.003 | 0.012 | 0.022 | 0.046 | 0.024 |
| Cr | 0.700 | 0.905 | 0.960 | 0.940 | 0.962 | 0.992 | 0.962 |  | 0.008 | 0.004 | 0.088 | 0.062 | 0.059 | 0.101 | 0.079 | 0.182 | 0.071 | 0.017 | 0.081 | 0.231 | 0.091 | 0.028 | 0.010 | 0.064 | 0.003 | 0.040 |
| Cu | 0.712 | 0.947 | 0.988 | 0.889 | 0.988 | 0.969 | 0.988 | 0.992 |  | 0.003 | 0.062 | 0.026 | 0.030 | 0.059 | 0.038 | 0.258 | 0.036 | 0.002 | 0.043 | 0.227 | 0.047 | 0.006 | 0.001 | 0.031 | 0.012 | 0.019 |
| Fe | 0.741 | 0.920 | 0.978 | 0.913 | 0.974 | 0.978 | 0.980 | 0.996 | 0.997 |  | 0.088 | 0.043 | 0.051 | 0.087 | 0.060 | 0.215 | 0.059 | 0.009 | 0.051 | 0.259 | 0.072 | 0.016 | 0.006 | 0.053 | 0.010 | 0.036 |
| Hg | 0.459 | 0.985 | 0.940 | 0.762 | 0.969 | 0.887 | 0.933 | 0.912 | 0.938 | 0.912 |  | 0.039 | 0.009 | 0.012 | 0.032 | 0.453 | 0.012 | 0.048 | 0.120 | 0.080 | 0.027 | 0.045 | 0.050 | 0.014 | 0.071 | 0.013 |
| In | 0.675 | 0.990 | 0.995 | 0.764 | 0.996 | 0.892 | 0.992 | 0.938 | 0.974 | 0.957 | 0.961 |  | 0.012 | 0.015 | 0.002 | 0.429 | 0.008 | 0.014 | 0.023 | 0.223 | 0.005 | 0.007 | 0.022 | 0.006 | 0.064 | 0.014 |
| K | 0.570 | 0.994 | 0.976 | 0.787 | 0.993 | 0.909 | 0.972 | 0.941 | 0.970 | 0.949 | 0.991 | 0.988 |  | 0.007 | 0.010 | 0.415 | 0.001 | 0.019 | 0.066 | 0.137 | 0.009 | 0.015 | 0.023 | 0.001 | 0.050 | 0.002 |
| Mg | 0.539 | **0.999** | 0.963 | 0.711 | 0.981 | 0.856 | 0.958 | 0.899 | 0.941 | 0.913 | 0.988 | 0.985 | 0.993 |  | 0.008 | 0.511 | 0.003 | 0.041 | 0.072 | 0.147 | 0.004 | 0.032 | 0.050 | 0.005 | 0.092 | 0.016 |
| Mn | 0.639 | 0.996 | 0.987 | 0.735 | 0.992 | 0.873 | 0.984 | 0.921 | 0.962 | 0.940 | 0.968 | **0.999** | 0.990 | 0.992 |  | 0.470 | 0.005 | 0.023 | 0.033 | 0.207 | 0.001 | 0.014 | 0.032 | 0.004 | 0.078 | 0.016 |
| Na | 0.607 | 0.500 | 0.638 | 0.959 | 0.630 | 0.869 | 0.647 | 0.819 | 0.742 | 0.785 | 0.547 | 0.571 | 0.585 | 0.489 | 0.530 |  | 0.448 | 0.300 | 0.416 | 0.517 | 0.494 | 0.337 | 0.273 | 0.433 | 0.191 | 0.366 |
| Ni | 0.579 | **0.998** | 0.978 | 0.760 | 0.992 | 0.891 | 0.973 | 0.929 | 0.964 | 0.941 | 0.988 | 0.992 | **0.999** | 0.997 | 0.995 | 0.552 |  | 0.023 | 0.058 | 0.152 | 0.004 | 0.017 | 0.029 | 0.000 | 0.063 | 0.006 |
| P | 0.698 | 0.964 | 0.994 | 0.861 | 0.995 | 0.955 | 0.993 | 0.983 | **0.998** | 0.991 | 0.952 | 0.986 | 0.981 | 0.959 | 0.977 | 0.700 | 0.977 |  | 0.035 | 0.215 | 0.031 | 0.001 | 0.001 | 0.019 | 0.020 | 0.011 |
| Pb | 0.813 | 0.938 | 0.987 | 0.744 | 0.967 | 0.862 | 0.988 | 0.919 | 0.957 | 0.949 | 0.880 | 0.977 | 0.934 | 0.928 | 0.967 | 0.584 | 0.942 | 0.965 |  | 0.373 | 0.043 | 0.026 | 0.046 | 0.053 | 0.098 | 0.065 |
| S | 0.106 | 0.840 | 0.740 | 0.691 | 0.803 | 0.784 | 0.729 | 0.769 | 0.773 | 0.741 | 0.920 | 0.777 | 0.863 | 0.853 | 0.793 | 0.483 | 0.848 | 0.785 | 0.627 |  | 0.194 | 0.223 | 0.208 | 0.158 | 0.188 | 0.139 |
| Sb | 0.611 | **0.998** | 0.980 | 0.718 | 0.988 | 0.861 | 0.976 | 0.909 | 0.953 | 0.928 | 0.973 | 0.995 | 0.991 | 0.996 | **0.999** | 0.506 | 0.996 | 0.969 | 0.957 | 0.806 |  | 0.020 | 0.040 | 0.004 | 0.088 | 0.017 |
| Se | 0.701 | 0.974 | **0.998** | 0.833 | **0.998** | 0.937 | 0.997 | 0.972 | 0.994 | 0.984 | 0.955 | 0.993 | 0.985 | 0.968 | 0.986 | 0.663 | 0.983 | **0.999** | 0.974 | 0.777 | 0.980 |  | 0.005 | 0.013 | 0.031 | 0.011 |
| Si | 0.691 | 0.956 | 0.988 | 0.882 | 0.991 | 0.967 | 0.988 | 0.990 | **0.999** | 0.994 | 0.950 | 0.978 | 0.977 | 0.950 | 0.968 | 0.727 | 0.971 | **0.999** | 0.954 | 0.792 | 0.960 | 0.995 |  | 0.024 | 0.012 | 0.013 |
| Sn | 0.593 | 0.997 | 0.982 | 0.771 | 0.995 | 0.899 | 0.978 | 0.936 | 0.969 | 0.947 | 0.986 | 0.994 | **0.999** | 0.995 | 0.996 | 0.567 | **0.999** | 0.981 | 0.947 | 0.842 | 0.996 | 0.987 | 0.976 |  | 0.058 | 0.004 |
| V | 0.646 | 0.913 | 0.953 | 0.940 | 0.962 | 0.994 | 0.954 | 0.997 | 0.988 | 0.990 | 0.929 | 0.936 | 0.950 | 0.908 | 0.922 | 0.809 | 0.937 | 0.980 | 0.902 | 0.812 | 0.912 | 0.969 | 0.988 | 0.942 |  |  |
| Zn | 0.588 | 0.986 | 0.979 | 0.824 | 0.994 | 0.933 | 0.976 | 0.960 | 0.981 | 0.964 | 0.987 | 0.986 | **0.998** | 0.984 | 0.984 | 0.634 | 0.994 | 0.989 | 0.935 | 0.861 | 0.983 | 0.989 | 0.987 | 0.996 | 0.997 |  |

Correlation coefficient values are below the diagonal, and significance values are above the diagonal.

**Table S2.** Loadings from Principal Component Analysis (PCA).

| **Loadings** | | | |
| --- | --- | --- | --- |
| Parameters | **PC 1** | **PC 2** | **PC 3** |
| Al | 0.14165 | **0.91739** | -0.10078 |
| As | **0.0025816** | -0.0029072 | 0.013173 |
| Au | **0.0043633** | 0.0011183 | 0.012379 |
| B | **0.0015952** | -0.0014624 | -0.0082316 |
| Ba | **0.0082534** | -0.0039084 | 0.024419 |
| Ca | **0.35547** | **-0.33056** | -0.68164 |
| Co | **0.0048248** | 0.0017872 | 0.012854 |
| Cr | **0.0019301** | -0.00079674 | -0.0011132 |
| Cu | **0.0018424** | -0.00039889 | 0.0018277 |
| Fe | **0.89483** | 0.029127 | 0.13992 |
| Hg | **0.004773** | -0.01115 | 0.018938 |
| In | **0.0027898** | -0.00051192 | 0.011092 |
| K | 0.024204 | -0.031703 | 0.087285 |
| Mg | 0.12326 | **-0.16635** | 0.64741 |
| Mn | 0.024197 | -0.010316 | 0.11258 |
| Na | 0.0051732 | -0.00027214 | -0.049987 |
| Ni | 0.0044792 | -0.005076 | 0.018823 |
| P | 0.0054181 | -0.0015526 | 0.0095077 |
| Pb | 0.006956 | 0.0098551 | 0.024723 |
| S | 0.018417 | **-0.11404** | 0.055349 |
| Sb | 0.003901 | -0.002492 | 0.019742 |
| Se | 0.011621 | -0.0019975 | 0.028109 |
| Si | 0.18867 | -0.077672 | 0.23526 |
| Sn | 0.0074111 | -0.0076363 | 0.029459 |
| V | **0.0014328** | -0.0013346 | -0.00068765 |
| Zn | 0.0084297 | -0.010524 | 0.023359 |

**Table S3.** Scores from Principal Component Analysis (PCA).

|  | **PC 1** | **PC 2** | **PC 3** |
| --- | --- | --- | --- |
| **P1** | 86.03 | 24.411 | -38.992 |
| **P2** | -359.58 | -54.038 | -0.85922 |
| **P3** | 449.25 | -26.123 | 16.077 |
| **P4** | -175.7 | 55.749 | 23.774 |

**Table S4.** Summary from Principal Component Analysis (PCA).

| **PC** | **Eigenvalue** | **%** **variance** |
| --- | --- | --- |
| **1** | 123130 | 97.454 |
| **2** | 2435.45 | 1.9276 |
| **3** | 781.585 | 0.6186 |
